# Best approximation of functions in generalized Hölder class

**DOI:** 10.1186/s13660-018-1864-y

**Published:** 2018-10-11

**Authors:** H. K. Nigam, Md. Hadish

**Affiliations:** Department of Mathematics, Central University of South Bihar, Gaya, India

**Keywords:** 41A10, 41A25, 42B05, 42A50, 40G05, 40C05, Best approximation, Generalized Hölder class, Matrix (*T*) means, $C^{1}$ means, $TC^{1}$ means, Fourier series, Conjugate Fourier series

## Abstract

Here, for the first time, error estimation of the functions $g\in H_{z}^{(w)}$ and $\tilde{g}\in H_{z}^{(w)}$ classes using $TC^{1}$ method of F. S. (Fourier Series) and C. F. S. (Conjugate Fourier Series), respectively, are determined. The results of (Dhakal in Int. Math. Forum 5(35):1729–1735, 2010; Dhakal in Int. J. Eng. Technol. 2(3):1–15, 2013; Kushwaha and Dhakal in Nepal J. Sci. Technol. 14(2):117–122, 2013) become the particular cases of our Theorem 2.1. Some important corollaries are also deduced from our main theorems.

## Introduction

Several results on the error estimation of a function *g* in Lipschitz and Hölder classes by a trigonometric polynomial using different single and product means have been obtained by the researchers like [1–11], and [12].

Our motivation for this work is to consider a more advanced class of functions that can provide best approximation by a trigonometric polynomial of degree not more than *r*. Therefore, in this work, we generalize the results of Kushwaha and Dhakal [3] and Dhakal [1, 2]. In fact, we obtain the results on the error estimation for the function $f\in H_{z}^{(w)}$ ($z\geq1$) by $T.C^{1}$ method by F. S. Thus, the results of Kushwaha and Dhakal [3] and Dhakal [1, 2] become the particulars cases of our Theorem 2.1.

We also obtain the results on the error estimation of the function $\tilde{g} \in H_{z}^{(w)}$ ($z\geq1$) by $T.C^{1}$ method of C. F. S.

Let “$T=(a_{r,m})$ be an infinite triangular matrix satisfying the conditions of regularity [13], i.e.,

1 $$ \begin{aligned} &\sum_{m=0}^{r}a_{r,m} =1 \quad \mbox{as } r\to\infty, \\ &a_{r,m} =0 \quad \mbox{for } m>r, \\ &\sum_{m=0}^{r}|a_{r,m}| \leq M, \quad \mbox{a finite constant}. \end{aligned} $$

The sequence-to-sequence transformation

2 $$ t_{r}^{T}:=\sum_{m=0}^{r}a_{r,m}s_{m}= \sum_{m=0}^{r}a_{r,r-m}s_{r-m} $$

defines the sequence $t_{r}^{T}$ of triangular matrix means of the sequence $\{s_{r}\}$ generated by the sequence of coefficients $(a_{r,m})$.

If $t_{r}^{T} \to s$ as $r\to\infty$, then the infinite series $\sum_{r=0}^{\infty}h_{r}$ or the sequence $\{s_{r}\}$ is summable to *s* by a triangular matrix (*T*-method) [14].”

“Let

3 $$\begin{aligned} C_{r}^{1} & = \frac{s_{0}+s_{1}+\cdots+s_{r}}{r+1} \\ &=\frac{1}{r+1}\sum_{m=0}^{r}s_{m} \to s \quad \mbox{as }r\to\infty. \end{aligned}$$

If $C_{r}^{1} \to s$ as $r\to\infty$, then the infinite series $\sum_{r=0}^{\infty}h_{r}$ is summable to *s* by $C^{1}$ means [14].” The $TC^{1}$ means (T-means of $C^{1}$ means) is given by

4 $$\begin{aligned} t_{r}^{T.C^{1}}&:=\sum_{m=0}^{r} a_{r,m} C_{m}^{1} \\ & = \sum_{m=0}^{r}a_{r,m} \frac{1}{m+1} \sum_{v=0}^{m} s_{m}. \end{aligned}$$

If $t_{r}^{T.C^{1}} \to s$ as $r \to\infty$, then the series $\sum_{r=0}^{\infty} h_{r}$ or the sequence $\{s_{r}\}$ is summable to *s* by $T.C^{1}$ means.

The regularity of *T* and $C^{1}$ methods implies the regularity of $T.C^{1}$ method.

### Remark 1

(Example)

Consider an infinite series

5 $$ 1+\sum_{n=1}^{\infty}(-1)^{n}.2n. $$

The *n*th partial sum of (5) is given by

$$s_{n}= \textstyle\begin{cases} n+1,&n\text{ is even}, \\ 0,&n\text{ is odd} \end{cases} $$

and so

$$C^{1}_{n}= \textstyle\begin{cases} 1, &n\text{ is even}, \\ 0, &n\text{ is odd}. \end{cases} $$

Therefore, series (5) is not summable by $(C,1)$ means.

If we take $a_{n,k}=\frac{1}{n+1}$, then series (5) is also not summable by *T* means. But series (5) is summable by $T.C^{1}$ means. So, the product means is more powerful than the individual means.

### Remark 2

$TC^{1}$ means reduces to

(i) $(H,\frac{1}{r+1})C^{1}$ or $H.C^{1}$ means if $a_{r,m}= \frac{1}{(r-m+1)\log(r+1)}$;
(ii) $(N,p_{r})C^{1}$ or $N_{p}C^{1}$ means if $a_{r,m}=\frac {p_{r-m}}{P_{r}}$, where $P_{r}=\sum_{m=0}^{r}p_{m} \neq0$;
(iii) $(N,p,q)(C,1)$ or $N_{p,q}C^{1}$ means if $a_{r,m}=\frac {p_{r-m} q_{m}}{R_{r}}$, where $R_{r}=\sum_{m=0}^{r}p_{m}q_{r-m}$;
(iv) $(\bar{N},p_{r})(C,1)$ or $\bar{N}_{p}C^{1}$ means if $a_{r,m}=\frac{p_{m}}{P_{r}}$.

Let $L^{z}[0,2\pi]= \{ g: [0,2\pi]\to\mathbb{R}: \int_{0}^{2\pi} |g(x)|^{z} \,dx <\infty, z\geq1\}$ be the space of functions (2*π*-periodic and integrable). We define the norm $\|\cdot\|_{(z)}$ by

$$\biggl\{ \frac{1}{2\pi} \int_{0}^{2\pi} \bigl\vert g(x) \bigr\vert ^{z}\,dx \biggr\} ^{\frac {1}{z}},\quad z\geq1. $$

As defined in “[14], $w:[0,2\pi] \to\mathbb{R}$ is an arbitrary function with $w(l)>0$ for $0< l\leq2\pi$ and $\lim_{l\to 0^{+}}w(l)=w(0)=0$.” Now we define

$$H_{z}^{(w)}= \biggl\{ g\in L^{z}[0,2\pi]:\sup _{l\neq0} \frac {\|g(\cdot,+l)-g(\cdot)\|_{z}}{w(l)} < \infty,z\geq1 \biggr\} $$

and

$$\Vert\cdot\Vert_{z}^{(w)}= \Vert g \Vert_{z}^{(w)}= \Vert g \Vert_{z}+ \sup_{l\neq0} \frac{\Vert g(\cdot+l)-g(\cdot)\Vert_{z}}{w(l)} ; \quad z\geq1. $$

### Note 1

$w(l)$ and $v(l)$ denote “Zygmund moduli of continuity [14].”

If we consider $\frac{w(l)}{v(l)}$ as positive and non-decreasing,

$$\Vert g \Vert_{z}^{(v)}\leq\max \biggl(1, \frac{w(2\pi)}{v(2\pi)} \biggr) \Vert g\Vert_{z}^{(w)} < \infty. $$

Thus,

$$H_{z}^{(w)}\subset H_{z}^{(v)}\subset L^{z} ;\quad z\geq1. $$

### Remark 3

(i) If $w(l)=l^{\alpha}$ in $H^{(w)}$, $H^{(w)}$ implies $H_{\alpha}$ class.
(ii) If $w(l)=l^{\alpha}$ in $H_{z}^{(w)}$, $H^{(w)}$ implies $H_{\alpha ,z}$ class.
(iii) If $z \to\infty$ in $H_{z}^{(w)}$, $H_{z}^{(w)}$ implies $H^{(w)}$ class and $H_{\alpha,z}$ class implies $H_{\alpha}$ class.

### Remark 4

We are not representing here the F. S. and C. F. S. as these trigonometric series are well known and the detailed work on these series can be found in [14].

We denote the *r*th partial sum of the F. S. as

$$s_{r}(g;x)-g(x)=\frac{1}{2\pi} \int_{0}^{\pi} \phi_{x}(l) \frac{\sin (r+\frac{1}{2})l}{\sin{\frac{l}{2}}}\,dl. $$

The *r*th partial sum of C. F. S. is defined as

$$s_{r}(\tilde{g};x)-\tilde{g}(x)=\frac{1}{2\pi} \int_{0}^{\pi} \psi _{x}(l) \frac{\cos(r+\frac{1}{2})l}{\sin(\frac{l}{2})}\,dl, $$

where

$$\tilde{g}=-\frac{1}{2\pi} \int_{0}^{\pi}\psi_{x}(l)\cot\biggl( \frac{l}{2}\biggr)\,dl. $$

“The error estimation of function *g* is given by

$$E_{r}(g)=\min\Vert g-t_{r}\Vert_{z}, $$

where $t_{r}$ is a trigonometric polynomial of degree *r* [14].”

We write

$$\begin{aligned}& \phi_{x}(l) =\phi(x,l)=g(x+l)+g(x-l)-2g(x), \\& \psi_{x}(l) =\psi(x,l)=g(x+l)-g(x-l), \\& \Delta p_{m} =p_{m}-p_{m+1},\quad m\geq{0}, \\& H_{r}(l) =\frac{1}{2\pi}\sum_{m=0}^{r}a_{r,m} \frac{1}{m+1}\sum_{v=0}^{m} \frac{\sin(v+\frac{1}{2})l}{\sin(\frac{l}{2})}, \\& \tilde{H}_{r}(l) =\frac{1}{2\pi}\sum_{m=0}^{r}a_{r,m} \frac{1}{m+1}\sum_{v=0}^{m} \frac{\cos(v+\frac{1}{2})l}{\sin(\frac{l}{2})}. \end{aligned}$$

## Main theorems

### Theorem 2.1

*If* $g\in H_{z}^{(w)}$ *class*; $z\geq1$ *and* $\frac{w(l)}{v(l)}$ *are positive and non*-*decreasing*, *then the error estimation of* *g* *by* $TC^{1}$ *means of F*.* S*. *is*

$$\bigl\Vert t_{r}^{T.C^{1}}-g \bigr\Vert _{z}^{(v)} =O \biggl( \frac{1}{r+1} \int _{\frac{1}{r+1}}^{\pi} \frac{w(l)}{l^{2}v(l)}\,dl \biggr), $$

*where* $T=(a_{r,m})$ *is an infinite triangular matrix satisfying* (1) *and* *w*, *v* *are defined as in Note* 1 *provided*

6 $$ \sum_{m =0}^{r-1}|\Delta a_{r,m}|=O \biggl(\frac{1}{r+1} \biggr) \quad \textit {and}\quad (r+1)a_{r,r}=O(1). $$

### Theorem 2.2

*If* $\tilde{g}\in H_{z}^{(w)}$ *class*; $z\geq1$ *and* $\frac{w(l)}{v(l)}$ *are positive and non*-*decreasing*, *then the error estimation of* *g̃* *by* $TC^{1}$ *means of C*.* F*.* S*. *is*

$$\bigl\Vert \tilde{t_{r}}^{T.C^{1}} -\tilde{g} \bigr\Vert _{z}^{(v)}=O \biggl( \frac{(\log (r+1)+1)}{r+1} \int_{\frac{1}{r+1}}^{\pi} \frac{w(l)}{l^{2}v(l)}\,dl \biggr), $$

*where* $T=(a_{r,m})$ *is an infinite triangular matrix satisfying* (1), (6) *and* *w*, *v* *are defined as in Note* 1.

## Lemmas

### Lemma 3.1

*Under condition* (1), $H_{r}(l)=O(r+1)$ *for* $0< l<\frac{1}{r+1}$.

### Proof

For $0< l<\frac{1}{r+1}$, $\sin(\frac{l}{2}) \geq\frac{l}{\pi}$, $\sin (r l) \leq r l$.

$$\begin{aligned}& H_{r}(l)=\frac{1}{2\pi} \sum_{m=0}^{r}a_{r,m} \frac{1}{m+1} \sum_{v=0}^{m} \frac{\sin(v+\frac{1}{2})l}{\sin(\frac{l}{2})}, \\& \begin{aligned} \bigl\vert H_{r}(l) \bigr\vert & \leq \frac{1}{2\pi} \times\frac{\pi}{l} \Biggl\vert \sum _{m=0}^{r}a_{r,m}\frac{1}{m+1}\sum _{v=0}^{m}\sin\biggl(v+\frac{1}{2} \biggr)l \Biggr\vert \\ & = \frac{1}{2l} \Biggl\vert \sum_{m=0}^{r}a_{r,m} \frac{1}{m+1} \sum_{v=0}^{m}\sin(2v+1) \frac{l}{2} \Biggr\vert \\ & \leq\frac{1}{2l} \Biggl\vert \sum_{m=0}^{r} a_{r,m} \frac{1}{m+1} \sum_{v=0}^{m}(2v+1) \frac{l}{2} \Biggr\vert \\ &=\frac{1}{4} \Biggl\vert \sum_{m=0}^{r}a_{r,m} \frac{1}{m+1}\sum_{v=0}^{m}(2v+1) \Biggr\vert \\ & = \frac{1}{4} \Biggl\vert \sum_{m=0}^{r} a_{r,m} \frac{1}{m+1} \times (m+1)^{2} \Biggr\vert \\ & = \frac{1}{4} \Biggl\vert \sum_{m=0}^{r}a_{r,m}(m+1) \Biggr\vert \\ & = \frac{1}{4}(m+1)\sum_{m=0}^{r}|a_{r,m}| \\ &=O(r+1). \end{aligned} \end{aligned}$$

 □

### Lemma 3.2

*Under conditions* (1) *and* (6), $H_{r}(l)=O ( \frac{1}{(r+1)l^{2}} )$ *for* $\frac{1}{r+1} \leq l \leq\pi$.

### Proof

For $\frac{1}{r+1} \leq l \leq\pi$, $\sin(\frac{l}{2}) \geq\frac {l}{\pi}$, $\sin^{2}{r}l \leq1$ and using Abel’s lemma, we have

$$\begin{aligned}& H_{r}(l) = \frac{1}{2\pi}\sum_{m=0}^{r}a_{r,m} \frac{1}{r+1} \sum_{v=0}^{r} \frac{\sin(v+\frac{1}{2})l}{\sin(\frac{l}{2})}, \\& \bigl\vert H_{r}(l) \bigr\vert \leq\frac{1}{2\pi} \times \frac{\pi}{l} \Biggl\vert \sum_{m=0}^{r}a_{r,m} \frac{1}{m+1} \sum_{v=0}^{m} \sin \biggl(v+\frac{1}{2}\biggr)l \Biggr\vert \\& \hphantom{ \vert H_{r}(l) \vert } = \frac{1}{2l} \Biggl\vert \sum _{m=0}^{r}a_{r,m} \frac{1}{m+1} \operatorname{Im} \Biggl\{ \sum_{v=0}^{m} e^{i(v+\frac{1}{2})l} \Biggr\} \Biggr\vert \\& \hphantom{ \vert H_{r}(l) \vert } = \frac{1}{2l} \Biggl\vert \sum _{m=0}^{r} a_{r,m} \frac{1}{m+1} \operatorname{Im} \Biggl\{ e^{i\frac{l}{2}} \sum_{v=0}^{m}e^{ivl} \Biggr\} \Biggr\vert \\& \hphantom{ \vert H_{r}(l) \vert } = \frac{1}{2l} \Biggl\vert \sum _{m=0}^{r} a_{r,m} \frac{1}{m+1} \operatorname{Im} \biggl\{ e^{\frac{il}{2}} \frac{1-e^{i(m+1)l}}{1-e^{il}} \biggr\} \Biggr\vert \\& \hphantom{ \vert H_{r}(l) \vert } = \frac{1}{2l} \Biggl\vert \sum _{m=0}^{r}a_{r,m} \frac{1}{m+1} \operatorname{Im} \biggl\{ \frac{e^{i(m+1)l}-1}{2i\sin(\frac{l}{2})} \biggr\} \Biggr\vert \\& \hphantom{ \vert H_{r}(l) \vert } \leq\frac{1}{2l} \times\frac{\pi}{l} \Biggl\vert \sum_{m=0}^{r} a_{r,m} \frac{1}{m+1} \sin^{2}(m+1) \frac{l}{2} \Biggr\vert \\& \hphantom{ \vert H_{r}(l) \vert } \leq\frac{\pi}{2l^{2}} \Biggl\vert \sum _{m=0}^{r} a_{r,m} \frac {1}{m+1} \Biggr\vert \\& \hphantom{ \vert H_{r}(l) \vert } = \frac{\pi}{2l^{2}} \Biggl\vert \sum _{m=0}^{r-1}(a_{r,m}-a_{r,m+1})\sum _{v=0}^{m}\frac{1}{v+1} +a_{r,r} \sum_{m=0}^{r} \frac{1}{m+1} \Biggr\vert \\& \hphantom{ \vert H_{r}(l) \vert } \leq \frac{\pi}{2l^{2}} \Biggl\vert \sum _{m=0}^{r-1} \Delta a_{r,m}\sum _{v=0}^{m}\frac{1}{v+1} \Biggr\vert + a_{r,r} \Biggl\vert \sum_{m=0}^{r} \frac {1}{m+1} \Biggr\vert \\& \hphantom{ \vert H_{r}(l) \vert } \leq\frac{\pi}{2l^{2}} \Biggl[ \sum _{m=0}^{r-1} |\Delta a_{r,m}| +a_{r,r} \Biggr] \max_{0\leq m \leq d} \Biggl\vert \sum _{m=0}^{d}\frac{1}{m+1} \Biggr\vert \\& \hphantom{ \vert H_{r}(l) \vert } =O \biggl( \frac{1}{(r+1)l^{2}} \biggr). \end{aligned}$$

 □

### Lemma 3.3

*Under condition* (1), $\tilde{H_{r}}(l)=O(\frac{1}{l})$ *for* $0< l<\frac{1}{r+1}$.

### Proof

For $0< l\leq\frac{1}{r+1}$, using $\sin(\frac{l}{2}) \geq\frac{l}{\pi }$ and $|\cos{rl}| \leq1$, we obtain

$$\begin{aligned}& \tilde{H_{r}}(l) =\frac{1}{2\pi} \sum _{m=0}^{r}a_{r,m} \frac{1}{m+1} \sum _{v=0}^{m} \frac{\cos(v+\frac{1}{2})l}{\sin(\frac{l}{2})}, \\& \begin{aligned} \bigl\vert \tilde{H_{r}}(l) \bigr\vert & \leq \frac{1}{2\pi} \times\frac{\pi}{l} \sum_{m=0}^{r} a_{r,m} \frac{1}{m+1} \sum_{v=0}^{m} \biggl|\cos\biggl(v+\frac{1}{2}\biggr)l\biggr| \\ & \leq\frac{1}{2l} \sum_{m=0}^{r} a_{r,m} \frac{1}{m+1} \sum_{v=0}^{m}1 \\ &\leq\frac{1}{2l} \sum_{m=0}^{r} a_{r,m}, \end{aligned} \\& \therefore\tilde{H_{r}}(l)= O \biggl( \frac{1}{l} \biggr). \end{aligned}$$

 □

### Lemma 3.4

*Under conditions* (1) *and* (6), $\tilde{H_{r}}(l)=O(\frac {1}{(r+1)l^{2}})$ *for* $\frac{1}{r+1} \leq l \leq\pi$.

### Proof

For $\frac{1}{r+1} \leq l \leq\pi$, using $\sin(\frac{l}{2}) \geq \frac{l}{\pi}$, Abel’s lemma, and $\vert \sum_{m=0}^{r}\frac{\sin (m+1)l}{m+1} \vert \leq1+\frac{\pi}{2}$ ∀*r* and *l* [15], we get

$$\begin{aligned} &\bigl\vert \tilde{H_{r}}(l) \bigr\vert \\ &\quad \leq\frac{1}{2\pi} \times\frac{\pi}{l} \Biggl\vert \sum_{m=0}^{r} a_{r,m} \frac{1}{m+1} \sum_{v=0}^{m} \cos\biggl(v+\frac {1}{2}\biggr)l \Biggr\vert \\ &\quad \leq\frac{1}{2l} \Biggl\vert \sum_{m=0}^{r} a_{r,m} \frac{1}{m+1} \biggl\{ \frac{2\sin(\frac{l}{2}) \cos\frac{l}{2}+ 2\sin(\frac{l}{2}) \cos \frac{3l}{2} +\cdots+ 2\sin(\frac{l}{2}) \cos(\frac{(2m+1)l}{2}) }{ 2\sin(\frac{l}{2})} \biggr\} \Biggr\vert \\ &\quad \leq\frac{1}{4l} \times\frac{\pi}{l} \Biggl\vert \sum _{m=0}^{r} a_{r,m} \frac{1}{m+1} \bigl\{ \sin{l} +\sin{2l} - \sin{l} + \sin{3l} - \sin{2l} + \cdots \\ &\qquad {}+ \sin{(m+1)l} -\sin{m l} \bigr\} \Biggr\vert \\ &\quad \leq\frac{\pi}{4l^{2}} \Biggl\vert \sum_{m=0}^{r} a_{r,m} \frac{\sin(m+1)l}{m+1} \Biggr\vert \\ &\quad \leq\frac{\pi}{4l^{2}} \Biggl\vert \sum_{m=0}^{r-1} (a_{r,m}-a_{r,m+1}) \sum_{v=0}^{m} \frac{\sin(v+1)l}{v+1} +a_{r,r} \sum_{m=0}^{r} \frac{\sin (m+1)l}{m+1} \Biggr\vert \\ &\quad \leq\frac{\pi}{4l^{2}} \Biggl[ \sum_{m=0}^{r-1}| \Delta a_{r,m}| \Biggl\vert \sum_{v=0}^{m} \frac{\sin(v+1)l}{v+1} \Biggr\vert +a_{r,r} \Biggl\vert \sum _{m=0}^{r} \frac{\sin(m+1)l}{m+1} \Biggr\vert \Biggr] \\ &\quad \leq \Biggl[ \frac{1}{l^{2}} \Biggl( \sum_{m=0}^{r-1}| \Delta a_{r,m}| +a_{r,r} \Biggr) \Biggr]. \\ &\quad = \biggl[\frac{1}{l^{2}} \biggl\{ O \biggl(\frac{1}{r+1} \biggr)+O \biggl(\frac{1}{r+1} \biggr) \biggr\} \biggr] \\ &\quad =O \biggl(\frac{1}{(r+1)l^{2}} \biggr). \end{aligned}$$

 □

### Lemma 3.5

(“([16], p. 93)”)

*Let* $g\in{H_{z}}^{(w)}$, *then for* $0< l\leq\pi$:

(i) $\Vert\phi(\cdot,l)\Vert_{z}=O(w(l))$;
(ii) $\Vert\phi(\cdot+y,l)-\phi(\cdot,l)\Vert_{z}= \scriptsize{\big\{\begin{array}{l} O(w(l)),\\ O(w(|y|)); \end{array}} $
(iii) *If* $w(l)$ *and* $v(l)$ *are defined as in Note* 1, *then* $\Vert\phi(\cdot+y,l)-\phi(\cdot,l)\Vert_{z}=O (v( \vert y \vert ) (\frac {w(l)}{v(l)} ) )$.

### Lemma 3.6

*Let* $\tilde{g}\in{H_{z}}^{(w)}$, *then for* $0< l\leq\pi$:

(i) $\Vert\psi(\cdot,l)\Vert_{z}=O(w(l))$;
(ii) $\Vert\psi(\cdot+y,l)-\psi(\cdot,l)\Vert_{z}= \scriptsize{\bigl\{\begin{array}{l} O(w(l)),\\ O(w(|y|)); \end{array}\bigr.} $
(iii) *If* $w(l)$ *and* $v(l)$ *are defined as in Note* 1, *then* $\Vert \psi(\cdot+y,l)-\psi(\cdot,l) \Vert _{z}=O (v( \vert y \vert ) (\frac {w(l)}{v(l)} ) )$.

### Proof

This lemma can be proved along the same lines as the proof of Lemma 3.5(iii). □

## Proof of the main theorems

### Proof of Theorem 2.1

#### Proof

Following Titchmarsh [17], $s_{r}(g;x)$ of F. S. is given by

$$ s_{r}(g;x)- g(x) = \frac{1}{2\pi} \int_{0}^{\pi} \phi_{x}(l) \frac {\sin(m+\frac{1}{2})l}{\sin(\frac{l}{2})} \,dl. $$

Now, denoting $T.C^{1}$ transform of $s_{r}(g;x)$ by ${t_{r}}^{T.C^{1}}$,

7 $$\begin{aligned}& \begin{aligned} {t_{r}}^{T.C^{1}}(x)- g(x) & = \sum _{m=0}^{r} a_{r,m} \bigl(C_{m}^{1}(x)- g(x) \bigr) \\ & = \sum_{m=0}^{r} a_{r,m} \Biggl( \frac{1}{m+1} \sum_{v=0}^{m} s_{v}(g;x)-g(x) \Biggr) \\ &= \int_{0}^{\pi} \phi_{x}(l) \Biggl( \frac{1}{2\pi} \sum_{m=0}^{r} a_{r,m} \frac{1}{m+1} \sum_{v=0}^{m} \frac{\sin(v+\frac{1}{2})l}{\sin (\frac{l}{2})} \Biggr) \,dl, \end{aligned} \\& {t_{r}}^{T.C^{1}}(x)- g(x) = \int_{0}^{\pi} \phi_{x}(l) {H_{r}}(l)\,dl. \end{aligned}$$

Let

8 $$ R_{r}(x)={t_{r}}^{T.C^{1}}(x)- g(x) = \int_{0}^{\pi} \phi_{x}(l) {H_{r}}(l)\,dl. $$

Then

$$ R_{r}(x+y)-R_{r}(x) = \int_{0}^{\pi} \bigl( \phi(x+y,l)-\phi(x,l) \bigr) {H_{r}(l)}\,dl. $$

“Using generalized Minkowski’s inequality Chui [18],” we get

9 $$\begin{aligned} \bigl\Vert R_{r}(\cdot,+y)-R_{r}(\cdot)\bigr\Vert _{z} & \leq \int_{0}^{\pi} \bigl\Vert \phi (\cdot+y,l)-\phi(\cdot,l) \bigr\Vert _{z} H_{r}(l)\,dt \\ &= \biggl( \int_{0}^{\frac{1}{r+1}} + \int_{\frac{1}{r+1}}^{\pi} \biggr) \bigl\Vert \phi(\cdot+y,l)-\phi( \cdot,l) \bigr\Vert _{z} H_{r}(l)\,dl \\ &=I_{1}+I_{2}. \end{aligned}$$

Using Lemmas 3.1 and 3.5(iii), we have

10 $$\begin{aligned} I_{1} &= \int_{0}^{\frac{1}{r+1}} \bigl\Vert \phi(\cdot+y,l)-\phi( \cdot,l) \bigr\Vert _{z} H_{r}(l)\,dl \\ &=O(r+1) \biggl( v\bigl( \vert y \vert \bigr) \int_{0}^{\frac{1}{r+1}} \frac{w(l)}{v(l)} \,dl \biggr) \\ &=O \biggl( v\bigl( \vert y \vert \bigr) \frac{w(\frac{1}{r+1})}{v(\frac{1}{r+1})} \biggr). \end{aligned}$$

Also, using Lemmas 3.2 and 3.5(iii), we get

11 $$\begin{aligned} I_{2} &= \int_{\frac{1}{r+1}}^{\pi} \bigl\Vert \phi(\cdot+y,l)-\phi( \cdot,l) \bigr\Vert _{z} H_{r}(l)\,dl \\ &=O \biggl(\frac{1}{r+1} \int_{\frac{1}{r+1}}^{\pi} v\bigl( \vert y \vert \bigr) \frac {w(l)}{l^{2}v(l)} \,dl \biggr). \end{aligned}$$

By (9), (10), and (11), we have

12 $$ \sup_{y \neq0} \frac{\Vert R_{r}(\cdot,+y)-R_{r}(\cdot)\Vert_{z}}{v(|y|)} =O \biggl( \frac{w(\frac{1}{r+1})}{v(\frac{1}{r+1})} \biggr) + O \biggl( \frac {1}{r+1} \int_{\frac{1}{r+1}}^{\pi} \frac{w(l)}{l^{2}v(l)} \,dl \biggr). $$

Again applying Minkowski’s inequality, Lemma 3.1, Lemma 3.2, and $\Vert \phi(\cdot,l) \Vert_{z}=O(w(l))$, we obtain

13 $$\begin{aligned} \bigl\Vert R_{r}(\cdot) \bigr\Vert _{z} &= \bigl\Vert t_{r}^{T.C^{1}}-g \bigr\Vert _{z} \\ &\leq \biggl( \int_{0}^{\frac{1}{r+1}}+ \int_{\frac{1}{r+1}}^{\pi} \biggr) \bigl\Vert \phi(\cdot,l) \bigr\Vert _{z} H_{r}(l)\,dl \\ &=O \biggl( (r+1) \int_{0}^{\frac{1}{r+1}} w(l) \,dl \biggr)+O \biggl( \frac {1}{r+1} \int_{\frac{1}{r+1}}^{\pi} \frac{w(l)}{l^{2}} \,dl \biggr) \\ &= O \biggl( w \biggl(\frac{1}{r+1} \biggr) \biggr)+O \biggl( \frac{1}{r+1} \int_{\frac{1}{r+1}}^{\pi} \frac{w(l)}{l^{2}} \,dl \biggr). \end{aligned}$$

Now, we have

14 $$ \bigl\Vert R_{r}(\cdot) \bigr\Vert _{z}^{v} = \bigl\Vert R_{r}(\cdot) \bigr\Vert _{z}+ \sup_{y\neq0} \frac{\Vert R_{r}(\cdot,+y)-R_{r}(\cdot)\Vert_{z}}{v(|y|)}. $$

Using (12) and (13), we get

15 $$\begin{aligned} \bigl\Vert R_{r}(\cdot) \bigr\Vert _{z}^{v} &= O \biggl( w \biggl(\frac{1}{r+1} \biggr) \biggr)+ O \biggl( \frac{1}{r+1} \int_{\frac{1}{r+1}}^{\pi} \frac {w(l)}{l^{2}} \,dl \biggr) \\ &\quad {}+ O \biggl( \frac{w (\frac{1}{r+1} )}{v ( \frac {1}{r+1} )} \biggr)+ O \biggl( \frac{1}{r+1} \int_{\frac {1}{r+1}}^{\pi} \frac{w(l)}{l^{2}v(l)} \,dl \biggr). \end{aligned}$$

By the monotonicity of $v(l)$, $w(l)=\frac{w(l)}{v(l)} v(l) \leq v(\pi ) \frac{w(l)}{v(l)}$ for $0< l\leq\pi$, we get

16 $$ \bigl\Vert R_{r}(\cdot) \bigr\Vert _{z}^{v} =O \biggl( \frac{w(\frac{1}{r+1})}{v(\frac {1}{r+1})} \biggr)+O \biggl( \frac{1}{r+1} \int_{\frac{1}{r+1}}^{\pi} \frac{w(l)}{l^{2}v(l)} \,dl \biggr). $$

Since *w* and *v* are moduli of continuity such that $\frac {w(l)}{v(l)}$ is positive and non-decreasing, therefore

$$ \frac{1}{r+1} \int_{\frac{1}{r+1}}^{\pi}\frac{w(l)}{l^{2}v(l)}\,dl \geq \frac{w(\frac{1}{r+1})}{v(\frac{1}{r+1})} \biggl(\frac{1}{r+1} \biggr) \int _{\frac{1}{r+1}}^{\pi} \frac{1}{l^{2}}\,dl \geq \frac{w ( \frac {1}{r+1} )}{2v ( \frac{1}{r+1} )}. $$

Then

17 $$ \frac{w ( \frac{1}{r+1} )}{v ( \frac{1}{r+1} )} =O \biggl( \frac{1}{r+1} \int_{\frac{1}{r+1}}^{\pi} \frac {w(l)}{l^{2}v(l)} \,dl\biggr). $$

From (16) and (17), we get

18 $$ \begin{aligned} & \bigl\Vert R_{r}(\cdot) \bigr\Vert _{z}^{(v)}=O \biggl(\frac{1}{r+1} \int_{\frac {1}{r+1}}^{\pi} \frac{w(l)}{l^{2}v(l)}\,dl \biggr), \\ & \bigl\Vert t_{r}^{T.C^{1}}-g \bigr\Vert _{z}^{(v)}=O \biggl(\frac{1}{r+1} \int _{\frac{1}{r+1}}^{\pi} \frac{w(l)}{l^{2}v(l)} \,dl\biggr). \end{aligned} $$

 □

### Proof of Theorem 2.2

#### Proof

The integral representation of $s_{r}(\tilde{g};x)$ is given by

$$ s_{r}(\tilde{g};x)- \tilde{g}(x) = \frac{1}{2\pi} \int_{0}^{\pi} \psi _{x}(l) \frac{\cos(r+\frac{1}{2})l}{\sin(\frac{l}{2})} \,dl. $$

Now, denoting $T.C^{1}$ transform of $s_{r}(\tilde{g};x)$ by $\tilde {t_{r}}^{T.C^{1}}$, we get

$$\begin{aligned}& \begin{aligned} \tilde{t_{r}}^{T.C^{1}}(x)- \tilde{g}(x) & = \sum _{m=0}^{r} a_{r,m} \bigl(C_{m}^{1}(x)- \tilde{g}(x) \bigr) \\ & = \sum_{m=0}^{r} a_{r,m} \Biggl( \frac{1}{m+1} \sum_{v=0}^{m} s_{v}(\tilde{g};x)-\tilde{g}(x) \Biggr) \\ &= \int_{0}^{\pi} \psi_{x}(l) \Biggl( \frac{1}{2\pi} \sum_{m=0}^{r} a_{r,m} \frac{1}{m+1} \sum_{v=0}^{m} \frac{\cos(v+\frac{1}{2})}{\sin (\frac{l}{2})} \Biggr) \,dl, \end{aligned} \\& \tilde{t_{r}}^{T.C^{1}}(x)- \tilde{g}(x) = \int_{0}^{\pi} \psi_{x}(l) \tilde{H_{r}}(l)\,dl. \end{aligned}$$

Let

$$ \tilde{R}_{r}(x) =\tilde{t}_{r}^{T.C^{1}}(x)- \tilde{g}(x)= \int_{0}^{\pi }\psi_{x}(l) \tilde{H}_{r} \,dl. $$

Then

$$ \tilde{R}_{r}(x+y)-\tilde{R}_{r}(x)= \int_{0}^{\pi} \bigl\{ \psi _{x}(x+y,l)- \psi_{x}(x,l) \bigr\} \tilde{H}_{r}(l)\,dl. $$

Using “generalized Minkowski’s inequality Chui [18],” we get

19 $$\begin{aligned} \bigl\Vert \tilde{R}_{r}(\cdot+y)-\tilde{R}_{r}(\cdot) \bigr\Vert _{z} & \leq \int_{0}^{\pi } \bigl\Vert \psi_{x}( \cdot+y,l) \bigr\Vert _{z} \tilde{H}_{r}(l)\,dl \\ &= \biggl( \int_{0}^{\frac{1}{r+1}}+ \int_{\frac{1}{r+1}}^{\pi} \biggr) \bigl\Vert \psi(\cdot+y,l)- \psi(\cdot,l) \bigr\Vert _{z} \tilde{R}_{r}(l)\,dl \\ &= I_{1}+I_{2}. \end{aligned}$$

Using Lemmas 3.3 and 3.6(iii), we have

20 $$\begin{aligned} I_{1} & = \int_{0}^{\frac{1}{r+1}} \bigl\Vert \psi(\cdot+y,l)-\psi( \cdot,l) \bigr\Vert _{z} \tilde{H_{r}}(l)\,dl \\ &= O \biggl( v\bigl( \vert y \vert \bigr) \frac{w(\frac{1}{r+1})}{ v(\frac{1}{r+1})} \int _{0}^{\frac{1}{r+1}} \frac{1}{l}\,dl \biggr) \\ &=O \biggl( v\bigl( \vert y \vert \bigr) \frac{w(\frac{1}{r+1})}{v(\frac{1}{r+1})} \log(r+1) \biggr). \end{aligned}$$

Again using Lemmas 3.4 and 3.6(iii), we have

21 $$\begin{aligned} I_{2} &= \int_{\frac{1}{r+1}}^{\pi} \bigl\Vert \psi(\cdot+y,l)-\psi( \cdot,l) \bigr\Vert _{z} \tilde{H_{r}}(l)\,dl \\ &=O \biggl( \frac{1}{r+1} \int_{\frac{1}{r+1}}^{\pi} v\bigl(|y|\bigr) \frac {w(l)}{l^{2} v(l)} \,dl \biggr). \end{aligned}$$

Using (19), (20), and (21), we have

22 $$ \sup_{y \neq0} \frac{\Vert\tilde{R}_{r}(\cdot+y)-\tilde{R}_{r}(\cdot) \Vert _{z}}{v(|y|)}= O \biggl( \frac{w(\frac{1}{r+1})}{v(\frac{1}{r+1})}\log (r+1) \biggr)+O \biggl( \frac{1}{r+1} \int_{\frac{1}{r+1}}^{\pi} \frac {w(l)}{l^{2} v(l)} \,dl \biggr). $$

Again applying Minkowski’s inequality, Lemma 3.3, Lemma 3.4, and $\Vert \psi(\cdot,l)\Vert_{z}= O(w(l))$, we have

23 $$\begin{aligned} \bigl\Vert \tilde{R_{r}}(\cdot) \bigr\Vert _{z} &= \bigl\Vert \tilde{t_{r}}^{T.C^{1}}-\tilde {g}\bigr\Vert _{z} \leq \biggl( \int_{0}^{\frac{1}{r+1}} + \int_{\frac{1}{r+1}}^{\pi } \biggr) \bigl\Vert \psi(\cdot,l)\bigr\Vert _{z} \tilde{H_{r}}(l)\,dl \\ &= O \biggl( \int_{0}^{\frac{1}{r+1}}\frac{w(l)}{l}\,dl \biggr)+ O \biggl( \frac{1}{r+1} \int_{\frac{1}{r+1}}^{\pi} \frac{w(l)}{l^{2}}\,dl \biggr) \\ & = O \biggl( w \biggl(\frac{1}{r+1} \biggr) \log(r+1) \biggr)+ O \biggl( \frac{1}{r+1} \int_{\frac{1}{r+1}}^{\pi} \frac{w(l)}{l^{2}}\,dl \biggr). \end{aligned}$$

Now, we have

$$ \bigl\Vert \tilde{R_{r}}(\cdot) \bigr\Vert _{z}^{(v)} = \bigl\Vert \tilde{R_{r}}(\cdot) \bigr\Vert _{z}+\sup _{y \neq0} \frac{ \Vert \tilde{R}_{r}(\cdot+y)-\bar{R}_{r}(\cdot) \Vert _{z}}{v(|y|)}. $$

Using (22) and (23), we get

$$\begin{aligned} \bigl\Vert \tilde{R_{r}}(\cdot) \bigr\Vert _{z}^{(v)}&= O \biggl( \bigl(\log(r+1)\bigr) w \biggl(\frac{1}{r+1} \biggr) \biggr)+O \biggl( \frac{1}{r+1} \int_{\frac {1}{r+1}}^{\pi} \frac{w(l)}{l^{2}}\,dl \biggr) \\ &\quad {} + O \biggl( \frac{w(\frac{1}{r+1})}{v(\frac{1}{r+1})} \log(r+1) \biggr)+O \biggl( \frac{1}{r+1} \int_{\frac{1}{r+1}}^{\pi} \frac {w(l)}{l^{2}v(l)}\,dl \biggr). \end{aligned}$$

By the monotonicity of $v(l)$, we have $w(l)=\frac{w(l)}{v(l)}v(l)\leq v(\pi) \frac{w(l)}{v(l)}$, $0< l\leq\pi$, we get

24 $$ \bigl\Vert \tilde{R_{r}}(\cdot) \bigr\Vert _{z}^{(v)}=O \biggl( \frac{w(\frac {1}{r+1})}{v(\frac{1}{r+1})} \log(r+1) \biggr)+ O \biggl( \frac{1}{r+1} \int_{\frac{1}{r+1}}^{\pi} \frac{w(l)}{l^{2}v(l)}\,dl \biggr). $$

Using the fact that $\frac{w(l)}{v(l)}$ is positive and non-decreasing, we have

$$\begin{aligned} \frac{1}{r+1} \int_{\frac{1}{r+1}}^{\pi} \frac{w(l)}{l^{2}v(l)}\,dl & \geq \frac{w(\frac{1}{r+1})}{v(\frac{1}{r+1})} \frac{1}{r+1} \int_{\frac {1}{r+1}}^{\pi} \frac{1}{l^{2}}\,dl \\ & \geq\frac{w(\frac{1}{r+1})}{2v(\frac{1}{r+1})}. \end{aligned}$$

Then

25 $$ \frac{w(\frac{1}{r+1})}{v(\frac{1}{r+1})} = O \biggl( \frac{1}{r+1} \int _{\frac{1}{r+1}}^{\pi} \frac{w(l)}{l^{2}v(l)}\,dl \biggr). $$

From (24) and (25), we get

26 $$ \begin{aligned} & \bigl\Vert \tilde{R_{r}}(\cdot) \bigr\Vert _{z}^{(v)}=O \biggl( \frac{\log(r+1)}{r+1} \int_{\frac{1}{r+1}}^{\pi} \frac{w(l)}{l^{2}v(l)} \,dl \biggr)+O \biggl( \frac{1}{r+1} \int_{\frac{1}{r+1}}^{\pi} \frac{w(l)}{l^{2}v(l)} \,dl \biggr), \\ &\therefore \bigl\Vert \tilde{t_{r}}^{T.C^{1}} -\tilde{g} \bigr\Vert _{z}^{(v)}= O \biggl( \frac{\log(r+1)+1}{r+1} \int_{\frac{1}{r+1}}^{\pi} \frac {w(l)}{l^{2}v(l)} \,dl \biggr). \end{aligned} $$

 □

## Corollary

### Corollary 5.1

*Let* $0 \leq\beta<\alpha\leq{1}$ *and* $\tilde{g} \in H_{(\alpha),z}$; $z\geq{1}$. *Then*

$$\bigl\Vert \tilde{t_{r}}^{T.C^{1}} -\tilde{g} \bigr\Vert _{(\beta),z}= \textstyle\begin{cases} O [ (\log(r+1)e)(r+1)^{\beta-\alpha} ]& \textit{if }0\leq {\beta}< \alpha< 1, \\ O [\frac{(\log(r+1)e)(\log(r+1) \pi)}{r+1} ] & \textit{if } \beta=0, \alpha=1. \end{cases} $$

### Proof

Putting $w(l)=l^{\alpha}$, $v(l)=l^{\beta}$, $0\leq\beta< \alpha\leq 1$ in (26)

$$\begin{aligned}& \bigl\Vert \tilde{t_{r}}^{T.C^{1}} -\tilde{g} \bigr\Vert _{(\beta),z} =O \biggl[ \frac{\log(r+1)e}{r+1} \int_{\frac{1}{r+1}}^{\pi} t^{\alpha-\beta-2} \,dl \biggr] \\& \quad \implies\quad \bigl\Vert \tilde{t_{r}}^{T.C^{1}} - \tilde{g} \bigr\Vert _{(\beta),z}= \textstyle\begin{cases} O ( \frac{(\log(r+1)e)}{(r+1)} \int_{\frac{1}{r+1}}^{\pi}l^{\alpha -\beta-2}\,dl )& \textit{if }0\leq{\beta}< \alpha< 1, \\ O ( \frac{\log(r+1)e}{r+1} \int_{\frac{1}{r+1}}^{\pi}l^{-1}\,dl ) & \textit{if }\beta=0, \alpha=1, \end{cases}\displaystyle \\& \therefore \bigl\Vert \tilde{t_{r}}^{T.C^{1}} -\tilde{g} \bigr\Vert _{(\beta),z}= \textstyle\begin{cases} O [ (\log(r+1)e)(r+1)^{\beta-\alpha} ]& \textit{if }0\leq {\beta}< \alpha< 1, \\ O [\frac{(\log(r+1)e)}{r+1} \times log(r+1)\pi ] & \textit{if } \beta=0, \alpha=1. \end{cases}\displaystyle \end{aligned}$$

 □

### Corollary 5.2

*Let* $0 \leq\beta<\alpha\leq{1}$, $a,b \in\mathbb{R}$ *and suppose* $w(l)=\frac{l^{\alpha}}{(\log\frac{1}{l})^{a}}$, $w(l)=\frac{l^{\beta }}{(\log\frac{1}{l})^{b}}$, $0< l\leq\pi$, $\tilde{g} \in H_{z}^{(w)}$, $z\geq{1}$. *Then*

$$\bigl\Vert \tilde{t_{r}}^{T.C^{1}} -\tilde{g} \bigr\Vert _{z}^{(v)}= \textstyle\begin{cases} O [ \frac{\log(r+1)e}{\{\log(r+1) \}^{b-a}} ]& \textit{if } \alpha=\beta \textit{ and } a-b \geq{-1}, \\ O [\frac{(\log(r+1)e)}{\log(r+1)} ] & \textit{if }\alpha =\beta \textit{ and } a-b=-1. \end{cases} $$

### Proof

We have

$$\begin{aligned}& \begin{aligned} \bigl\Vert \tilde{t_{r}}^{T.C^{1}} - \tilde{f} \bigr\Vert _{z}^{(v)}&=O \biggl( \frac {\log(r+1)e}{r+1} \int_{\frac{1}{r+1}}^{\pi} \frac{l^{\alpha }}{l^{2}(\log\frac{1}{l})^{a} \times\frac{l^{\beta}}{(\log\frac {1}{l})^{b}}}\,dl \biggr) \\ &= O \biggl( \frac{\log(r+1)e}{r+1} \int_{\frac{1}{r+1}}^{\pi} l^{\alpha -\beta-2} \biggl(\log \frac{1}{l}\biggr)^{b-a}\,dl \biggr) \end{aligned} \\& \therefore \bigl\Vert \tilde{t_{\eta}}^{T.C^{1}} -\tilde{g} \bigr\Vert _{z}^{(v)}= \textstyle\begin{cases} O [ \frac{\log(r+1)e}{\{\log(r+1) \}^{b-a}} ]& \text{if $\alpha=\beta\mbox{ and } a-b \geq{-1} $}.\\ O [\frac{(\log(r+1)e)}{\log(r+1)} ] & \text{if $\alpha =\beta\mbox{ and }a-b=-1$}. \end{cases}\displaystyle \end{aligned}$$

 □

### Corollary 5.3

*If* $a_{r,m}= \frac{1}{(r-m+1)\log(r+1)}$, *then* $T.C^{1}$ *means reduces to* $(H, \frac{1}{r+1} )(C,1)$ *means and error estimation of a function* $g\in H_{z}^{(w)}$ *by* $(H, \frac{1}{r+1})(C,1)$ *means of F*.* S*. *is*

$$\bigl\Vert t_{r}^{H.C^{1}}-g \bigr\Vert _{z}^{(v)}=O \biggl( \frac{1}{r+1} \int _{\frac{1}{r+1}}^{\pi} \frac{w(l)}{l^{2}v(l)}\,dl \biggr). $$

### Corollary 5.4

*If* $a_{r,m}=\frac{p_{r-m}}{P_{r}}$, *then* $T.C^{1}$ *means reduces to* $N_{p}.C^{1}$ *and the error estimation of* $g \in H_{v}^{(w)}$ *by* $N_{p}.C^{1}$ *means of F*.* S*. *is*

$$\bigl\Vert t_{r}^{N_{p}.C^{1}}-g \bigr\Vert _{z}^{(v)}=O \biggl( \frac{1}{r+1} \int _{\frac{1}{r+1}}^{\pi} \frac{w(l)}{l^{2}v(l)}\,dl \biggr). $$

### Corollary 5.5

*If* $a_{r,m}=\frac{p_{r-m}q_{m}}{R_{r}}$, *then* $T.C^{1}$ *means reduces to* $N_{p,q}.C^{1}$ *and the error estimation of* $g \in H_{v}^{(w)}$ *by* $N_{p,q}.C^{1}$ *means of F*.* S*. *is*

$$\bigl\Vert t_{r}^{N_{p,q}.C^{1}}-g \bigr\Vert _{z}^{(v)}=O \biggl( \frac{1}{r+1} \int_{\frac{1}{r+1}}^{\pi} \frac{w(l)}{l^{2}v(l)}\,dl \biggr). $$

### Corollary 5.6

*If* $a_{r,m}= \frac{1}{(r-m+1)\log(r+1)}$, *then* $T.C^{1}$ *means reduces to* $(H, \frac{1}{r+1} )(C,1)$ *means and the error estimation of a function* $\tilde{g}\in H_{z}^{(w)}$ *by* $(H, \frac{1}{r+1})(C,1)$ *means of C*.* F*.* S*. *is*

$$\bigl\Vert \tilde{t_{r}}^{H.C^{1}}-\tilde{g} \bigr\Vert _{z}^{(v)}=O \biggl( \frac {(\log(r+1)+1)}{r+1} \int_{\frac{1}{r+1}}^{\pi} \frac {w(l)}{l^{2}v(l)}\,dl \biggr). $$

### Corollary 5.7

*If* $a_{r,m}=\frac{p_{r-m}}{P_{r}}$, *then* $T.C^{1}$ *means reduces to* $N_{p}.C^{1}$ *and the error estimation of* $\tilde{g} \in H_{v}^{(w)}$ *by* $N_{p}.C^{1}$ *means of C*.* F*.* S*. *is*

$$\bigl\Vert \tilde{t_{r}}^{N_{p}.C^{1}}-\tilde{f} \bigr\Vert _{z}^{(v)}=O \biggl( \frac{(\log(r+1)+1)}{r+1} \int_{\frac{1}{r+1}}^{\pi} \frac {w(l)}{l^{2}v(l)}\,dl \biggr). $$

### Corollary 5.8

*If* $a_{r,m}=\frac{p_{r-m}q_{m}}{R_{r}}$, *then* $T.C^{1}$ *means reduces to* $N_{p,q}.C^{1}$ *and the error estimation of* $\tilde{f} \in H_{v}^{(w)}$ *by* $N_{p,q}.C^{1}$ *means of C*.* F*.* S*. *is*

$$\bigl\Vert \tilde{t_{r}}^{N_{p,q}.C^{1}}-\tilde{g} \bigr\Vert _{z}^{(v)}=O \biggl( \frac{(\log(r+1)+1)}{r+1} \int_{\frac{1}{r+1}}^{\pi} \frac {w(l)}{l^{2}v(l)}\,dl \biggr). $$

### Remark 5

(i) If $z\to\infty$ in $H_{z}^{(w)}$ class, then $H_{z}^{(w)}$ class reduces to $H^{(w)}$ class. Also putting $w(l)=l^{\alpha}$ and $v(l)=l^{\beta}$ in our Theorem 2.1, $H^{(w)}$ class reduces to $H_{\alpha}$ class; then, by putting $\beta=0$ in $H_{\alpha}$ class, $H_{\alpha}$ class reduces to Lip*α* class.
(ii) In our Theorem 2.1, by putting $w(l)=l^{\alpha}$, $v(l)=l^{\beta}$ in $H_{z}^{(w)}$ class, $H_{z}^{(w)}$ class reduces to $H_{\alpha,z}$; then, by putting $\beta=0$ in $H_{\alpha,z}$ class, $H_{\alpha,z}$ class reduces to $\operatorname{Lip}(\alpha,z)$ class.

## Particular cases

6.1. Using Remark 4(i), our Theorem 2.1 becomes a particular case of Dhakal [1].
6.2. Using Remark 4(ii) and putting $a_{r,m}=\frac {p_{r-m}q_{m}}{R_{r}}$, where $R_{r}=\sum_{m=0}^{r}p_{\mu}q_{r-m}$ in our of Theorem 2.1, our result of Theorem 2.1 becomes a particular case of the main theorem of Kushwaha and Dhakal [3].
6.3. Using Remark 4(i) and putting $a_{r,m}=\frac {p_{r-m}q_{m}}{R_{r}}$, where $R_{r}=\sum_{m=0}^{r}p_{m}q_{r-m}$ in our Theorem 2.1, our Theorem 2.1 becomes a particular case of the main theorem of Dhakal [2].

## Conclusion

Approximation by trigonometric polynomials is at the heart of approximation theory. Much of the advances in the theory of trigonometric approximation are due to the periodicity of the functions. The study of error approximation of periodic functions in Lipschitz and Hölder classes has been of great interest among the researchers [1–11], and [12] in recent past. The trigonometric Fourier approximation (TFA) is of great importance due to its wide applications in different branches of engineering such as electronics and communication engineering, electrical and electronics engineering, computer science engineering, etc. Several elegant results on TFA can be found in a monograph [14].

In this paper, we, for the first time, obtain the best approximation of the functions *g* and *g̃* in a generalized Hölder class $H_{r}^{(w)}$ ($r\geq1$) using Matrix-$C^{1}$ $(T.C^{1})$ method of F. S. and C. F. S. respectively. Since, in view of Remark 2, the product summability means $H.C^{1}$, $N_{p}C^{1}$, $N_{p,q}C^{1}$, and $\bar{N}_{p}C^{1}$ are the particular cases of Matrix-$C^{1}$ method, so our results also hold for these methods, which are represented in a form of corollaries. In view of Remark 1, it has been shown that $(TC^{1})$ method is more powerful than the individual *T* method and $C^{1}$ method. Moreover, in view of Remark 5, some previous results (see Sect. 6) become the particular cases of our Theorem 2.1. We also deduce a corollary for the $H_{\alpha ,r}$ class ($r\geq1$).

Some other studies regarding the modulus of continuity (smoothness) of functions using more generalized functional spaces may be addressed as a future work.
